# Radiosensitivity and Induction of Apoptosis by High LET Carbon Ion Beam and Low LET Gamma Radiation: A Comparative Study

**DOI:** 10.1155/2014/438030

**Published:** 2014-06-15

**Authors:** Atanu Ghorai, Nitai P. Bhattacharyya, Asitikantha Sarma, Utpal Ghosh

**Affiliations:** ^1^Department of Biochemistry & Biophysics, University of Kalyani, Kalyani 741235, India; ^2^Crystallography and Molecular Biology Division, Saha Institute of Nuclear Physics, 1/AF Bidhannagar, Kolkata 700 064, India; ^3^Inter-University Accelerator Center (IUAC), Aruna Asaf Ali Marg, New Delhi 110067, India

## Abstract

Cancer treatment with high LET heavy ion beam, especially, carbon ion beam (^12^C), is becoming very popular over conventional radiotherapy like low LET gamma or X-ray. Combination of Poly(ADP-ribose) polymerase (PARP) inhibitor with xenotoxic drugs or conventional radiation (gamma or X-ray) is the newer approach for cancer therapy. The aim of our study was to compare the radiosensitivity and induction of apoptosis by high LET ^12^C and low LET gamma radiation in HeLa and PARP-1 knocked down cells. We did comet assay to detect DNA breaks, clonogenic survival assay, and cell cycle analysis to measure recovery after DNA damage. We measured apoptotic parameters like nuclear fragmentation and caspase-3 activation. DNA damage, cell killing, and induction of apoptosis were significantly higher for ^12^C than gamma radiation in HeLa. Cell killing and apoptosis were further elevated upon knocking down of PARP-1. Both ^12^C and gamma induced G_2_/M arrest although the ^12^C had greater effect. Unlike the gamma, ^12^C irradiation affects DNA replication as detected by S-phase delay in cell cycle analysis. So, we conclude that high LET ^12^C has greater potential over low LET gamma radiation in killing cells and radiosensitization upon PARP-1 inhibition was several folds greater for ^12^C than gamma.

## 1. Introduction

Tumor killing by high linear energy transfer (LET) heavy ion beam like carbon ion beam (^12^C) is becoming one of the promising ways especially for radioresistant tumor cells for its higher relative biological effectiveness compared with conventional low LET radiation like X-ray, gamma ray, and so forth. Ionising radiation kills cells by producing DNA breaks followed by induction of DNA damage induced apoptosis or necrosis. The quality and quantity of DNA breaks produced by low and high LET radiation are different. The density of single strand break (SSB) produced by low LET radiation is low in a particular region of DNA. This SSB can produce double strand break (DSB) when two SSBs are in two opposite strands in a close proximity. High LET radiation like carbon ion beam (^12^C) produces huge number of SSBs breaks in a small region of DNA; called clustered DNA damage and this can lead to DSBs. Therefore, DSBs are high for high LET radiation whereas SSBs are mostly observed for low LET radiation. So, the mechanism of DNA repair after high and low LET radiation is most likely different and a different set of proteins are involved in two distinct repair processes. One of the well-known SSB repairs is base excision repair (BER) where PARP-1 is involved [1–3]. Generally DSB repair is done by homologous recombination and non-homologous end joining [4, 5]. PARP-1 is involved in the DNA replication process and backup non-homologous end joining, so PARP-1 is most likely involved in DSB repair also [6, 7]. There will be DNA damage induced apoptosis if cells fail to repair DNA after exposure with high or low LET radiation. A large number of reports showed that gamma or ^12^C irradiation induced apoptosis in various cell types [8–14]. PARP-1 plays dual role in apoptosis [15]. Inhibition of PARP-1 can induce apoptosis [16, 17] or decrease apoptosis [18–20]. Cell exposed to ionizing radiation produces DNA breaks after which the immediate response is activation of PARP-1 that leads to depletion of NAD^+^ and ATP store inside the cells. Severe DNA damage leads to overactivation of PARP-1, which causes extreme depletion of energy sources followed by the necrotic cell death [21, 22]. Several diseases like stroke, myocardial infarction, and mesenteric ischemia reperfusion are characterized predominantly by necrotic cell death caused by overactivation of PARP-1 [23]. In such cases use of pharmacological inhibitors of PARP-1 prevents the cells from necrotic death [24].

PARP-1 null mice were developed independently in different laboratories and showed hypersensitivity to alkylating agent and gamma radiation [25–29]. But none of the PARP-1 null mice showed induction of apoptosis in it. We observed earlier that PARP inhibition by benzamide induced apoptosis [30, 31]. Pharmacological inhibitors of PARP-1 have long been used as chemosensitizer or radiosensitizer since PARP-1 is involved in DNA repair [32–35]. A large number of encouraging preclinical data showed that treatment with PARP inhibitors in combination with DNA damaging agents or gamma radiation potentiates the tumor cell killing [36–39]. Inhibition of PARP by ABT-888 enhanced breast cancer cell senescence both in vitro and in vivo [16]. PARP-1 inhibitor GPI-15427 induced significant sensitization to radiotherapy of head and neck squamous cell carcinoma by enhancing apoptosis [17]. PARP inhibitor E7016 showed higher radiosensitivity in vitro and in vivo through the inhibition of DNA repair in glioblastoma cells although status of apoptosis was not mentioned [40]. To avoid the nonspecific effect of pharmacological inhibitors of PARP-1 if any, one should get better result if the inhibition of PARP-1 is obtained by siRNA. We have observed earlier that inhibition of PARP-1 by pharmacological inhibitor and/or siRNA for PARP-1 resulted in induction of apoptosis in undamaged cells [31], implicating antiapoptotic role of PARP-1. There is as such no report of sensitization and apoptosis induction by knocking down of PARP-1 in cells treated with carbon ion beam. Here, we have compared radiosensitization and induction of apoptosis by high LET carbon ion beam and low LET gamma radiation in HeLa and siPARP-1 HeLa (HsiI) cells.

## 2. Materials and Methods

### 2.1. Cell Culture

Human cervical cancer cell line HeLa was obtained from the National Centre for Cell Sciences, Pune, India. HeLa cells were grown in MEM supplemented with 10% fetal bovine serum (complete medium) at 37°C in humidified atmosphere containing 5% CO_2_.

### 2.2. Irradiation with Carbon Ion Beam

The irradiation was carried out at the dedicated Radiation Biology Beam line of the 15UD Pelletron of Inter-University Accelerator Center (IUAC), New Delhi, India. In this experiment, 62 MeV [5.2 MeV/u] ^12^C beam with corresponding entrance LET 290 keV/*μ*m was used. The cells were irradiated in a sterilized closed chamber which is a computer controlled automated irradiation system called ASPIRE installed at the Radiation Biology Beam Line. The system has the facility to place the 35 mm petri dishes in front of a 40 mm diameter window in order to be irradiated by the heavy ion beam having flux of about 2 × 10^5^ particles/cm^2^/sec. The petri dishes without the lead were stacked and immersed in a tank filled with serum-free medium during the entire duration of irradiation. The dishes were picked up remotely one by one for irradiation with preassigned dose and after exposure the plate was reimmersed back into the tank containing medium. The radiation field uniformity was better than 95% as ascertained during prior experiments using solid state nuclear track detectors. All the samples were irradiated within 13–18 h growth condition and after irradiation fresh complete medium was added into plates and incubated for the time period as required in the specific experiments. The dosimetry is done using the particle counts from a pair of silicon surface barrier detectors, out of which one is placed at the atmosphere in the exact sample position and the other inside beam line as a monitor. For measuring the particle fluence online as well as controlling the irradiation protocol, the monitor detector is calibrated prior against the counts from the detector placed at the sample position. The dose in Gray (Gy) is calculated from the particle fluence using the relation

(1) Dose [Gy]=1.6×10−9  ×LET [keV/μm]×Fluence [particles/cm2]  

see [41].

### 2.3. Gamma Treatment

The cells were irradiated with various doses of gamma ray [Co^60^] at Saha Institute of Nuclear Physics, Kolkata, India. The dose rate was 57 rad/min. We used different doses at the range of 0–4 Gy. After irradiation immediately medium was replaced with fresh complete medium and incubated for 24 h as required for the experiments done.

### 2.4. Preparation of PARP-1 Knocked Down HeLa (HsiI) Cells

PARP-1 knocked down HeLa cells were prepared by transfection of HeLa cells with or without siRNA insert for PARP-1 in vector plasmid pRNA-U6.1 using Lipofectamine 2000 following methods provided by the manufacturer (Invitrogen, USA) and prepared HsiI and H-vector (negative control) cells as described in our earlier studies [31, 42].

### 2.5. Reverse-Transcriptase (RT) PCR

To check the PARP-1 gene silencing in PARP-1 knocked down (HsiI) cells, reverse transcriptase PCR (RT-PCR) was performed using PARP-1 gene-specific primer as followed in our earlier procedure [43]. The primers used for PARP-1 were 5′-CGTGTGGGTACGGTGA-3′ (forward) and 5′-GGCCATAGTCAATCTCAA-3′ (reverse) and for *β*-actin were 5′-TCCTGTGGCATCCACGAAACT-3′ (forward) and 5′-GAAGCATTTGCGGTGGAC-3′ (reverse).

### 2.6. NAD Assay

NAD assay was performed according to the method described in our earlier studies [42, 43].

### 2.7. Comet Assay

DNA damage was measured using the alkaline single cell gel electrophoresis assay [44] with some modification. In brief, normal HeLa and HsiI cells were exposed to ^12^C ion beam or gamma at different doses (varied from 0 to 4 Gy) and allowed to grow at 37°C for 24 h. Washed twice with phosphate buffered saline (PBS), ~1 × 10^4^cells were suspended in 0.6% low melting agarose and layered over a frosted microscopic slide previously coated with a layer of 0.75% (w/v) normal melting agarose. The slides were then immersed in a lysing solution of pH 10 (NaCl 2.5 M, Na_2_-EDTA 0.1 M, Tris 10 mM, and NaOH 0.3 M) and left overnight. The slides were then transferred to a horizontal electrophoresis chamber containing alkaline solution (300 mM NaOH, 1 mM Na_2_-EDTA) and allowed to soak for 20 minutes for unwinding of DNA. Electrophoresis was then carried out for 20 min (300 mA, 20 V). Slides were then washed thrice with neutralizing buffer (Tris 0.4 M, pH 7.5), stained with ethidium bromide (final concentration 40 *μ*g/mL), and observed under a Zeiss fluorescent microscope. DNA damage was quantified by tail moment measurement, calculated by multiplying the total intensity of the comet tail by the tail length measuring from the centre of the comet head. 200 cells were counted randomly for each slide and it was done for three times for each dose of each cell type.

### 2.8. Clonogenic Cell Survival Assay

Cell death was measured by colony forming ability of adherent cells. After irradiation both cells HeLa and PARP-1 knocked down HeLa (HsiI) were detached by trypsin, counted by Countess (Invitrogen, USA) or under haemocytometer (SIGMA), serially diluted, and finally plated at a density of 300 cells/tissue culture petri dishes (60 mm). For each dose, cells were seeded into 4 plates each with 4 mL of growth medium and allowed to grow for 10 days at 37°C in humidified CO_2_ incubator. After 10 days of growth the visible colonies (approximately 50 cells in each colony) were fixed and stained with 50% ethanol having 0.25% methylene blue for at least 30 min and visually counted. Surviving fraction was calculated by dividing the number of colonies found in irradiated samples by the number of seeded cells and the plating efficiencies of the unirradiated control cells of each cell type.

### 2.9. Cell Cycle Analysis by FACS

Cell cycle analysis was done following our earlier studies [45–47] with slight modifications. In brief, after treatment with different doses (0–4 Gy) of ^12^C ion beam or gamma radiation followed by 24 h of postirradiation incubation, cells were trypsinized and washed twice with cold phosphate buffered saline (PBS). Now cells were fixed in chilled 70% ethanol in PBS for 2 h at 4°C. After fixation, cells were washed thrice with cold PBS and then stained with a solution containing 10 *μ*g/mL propidium iodide (PI), 100 *μ*g/mL of DNase-free RNase A, and 0.1% (v/v) Triton X-100 in the dark for 15 min at room temperature before flow cytometric analysis. Now, the nuclei of each cell were become labelled with PI and the PI fluorescence of individual nuclei was determined by a fluorescence-activated cell sorter (FACS Calibur, BD Bioscience, USA) using the blue laser of 488 nm excitation. Data acquisition was done using CellQuestPro software (Becton Dickinson) and for each sample 20,000 cells were taken. The percentages of cells in each phase of the cell cycle was analyzed using ModFit LT software (Variety Software) supplied by BD Bioscience, USA.

### 2.10. Detection of Nuclear Fragmentation

Detection of nuclear fragmentation was done as reported earlier [30, 45] with some modification. Briefly, HeLa cells as well as PARP-1 knocked down cells (HsiI) were grown over cover slips inside the 35 mm or 60 mm culture plates overnight and then treated with ^12^C ion beam or gamma radiation at different doses (varied from 0 to 4 Gy) followed by further 24 h incubation. Then the cover slips were washed twice with PBS and the cells were fixed using 1 mL of fixative solution of methanol : acetone (1 : 1) for 1 h in 4°C condition. Again the cells were washed thrice with PBS. Finally it was stained with Hoechst 33258 dye (1 *μ*M), incubated for 5 min in dark, and washed with PBS. Cells were examined under fluorescence microscope (Carl Zeiss) using the appropriate filter. Apoptotic cells were distinguished by fragmented nucleus, chromatin condensation and formation of membrane bound apoptotic bodies. At least 300 of apoptotic cells as well as the normal cells were randomly counted and percentage of apoptotic cells was calculated at each dose.

### 2.11. Caspase-3 Activation

Caspase-3 activity assays were performed according to the protocols recommended by the manufacturer (ApoAlert Caspase 3 Fluorescence Assay Kit and ApoAlert Caspase 3 Colorimetric Assay Kit from Clontech, USA). In short, after treatment with different doses (0–4 Gy) of carbon ion beam and gamma radiation cells were grown for further 24 h. After trypsinization, cells were counted by Countess (Invitrogen, USA). Two million cells were taken, washed with cold PBS, and pelleted down. Then cells were lysed in 50 *μ*L of chilled lysis buffer (as provided by the respective manufacturer) on ice for 10 min. The lysate was centrifuged at 18,000 g for 5 min at 4°C to precipitate cell debris and supernatant was collected. 50 *μ*L of 2X reaction buffer containing 10 mM DTT was added to it. Five *μ*L of reconstituted Ac-DEVD-AFC (for fluorimetric assay) and DEVD-pNA (for colorimetric assay), the substrates for caspase-3 (1 mM), was added to each reaction mix and allowed to incubate for 1 h at 37°C water bath. The fluorescence of liberated AFC was measured in Spectrofluorimeter with an excitation at 400 nm and an emission at 494 nm whereas the absorbance of liberated free pNA was measured in Spectrophotometer at 405 nm. To check the enzyme-substrate specificity, the inhibitors of the caspase-3 (supplied by the manufacturer in the assay kits) were used together with their respective substrates during the assay and found inhibited by the inhibitors showing the specificity of the assays (data not shown).

### 2.12. Statistical Calculation

Each experiment was repeated at least 3 times and 2-tailed paired-samples* t*-test was performed using SPSS Software (version 21) at the same doses of both carbon ion beam and gamma radiation in both cell types—HeLa and HsiI. The significance values were denoted as “∗” (0.01 < *P* ≤ 0.05), “∗∗” (0.001 < *P* ≤ 0.01), and “∗∗∗” (*P* ≤ 0.001). Statistical analysis was also done for the irradiated samples of each cell type with respect to the unirradiated control of each cell type using one-way ANOVA with Dunnett's test.

## 3. Results and Discussion

### 3.1. Expression and Activity of PARP-1 in HsiI Cells Compared with Parental HeLa Cells

**Figure 1:**
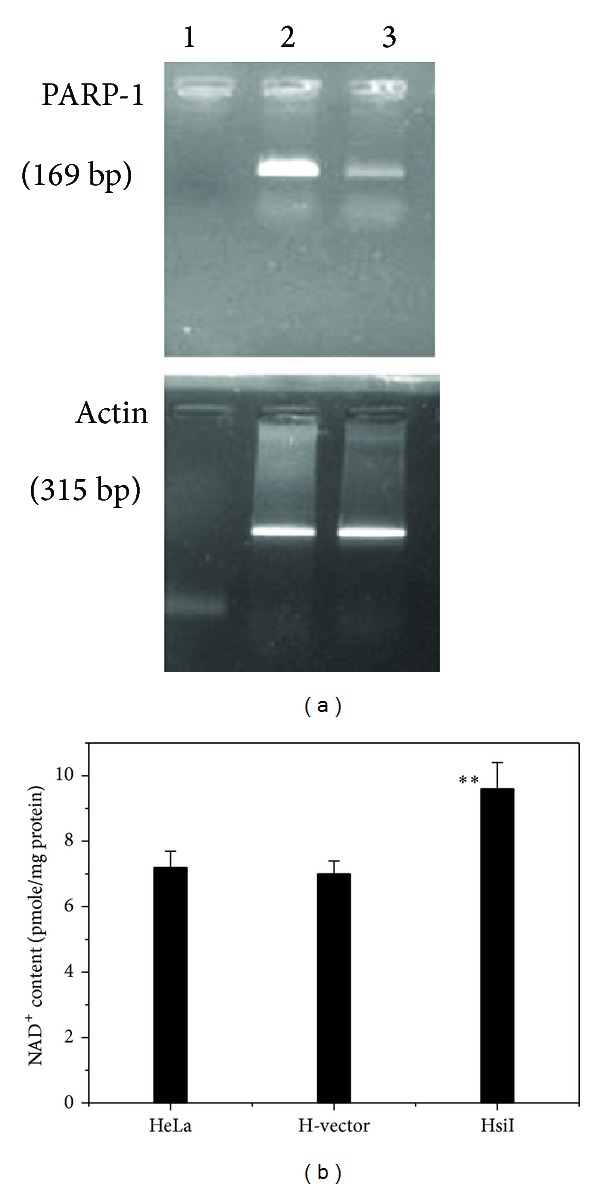
(a) RT-PCR for checking PARP-1 gene silencing. RT-PCR products for PARP-1 (upper panel) and beta actin (lower panel). Lanes 1, 2, and 3 represent negative control, HeLa, and HsiI, respectively. (b) Bar diagram for NAD^+^ content (pmole/mg protein) in HeLa, H-vector, and HsiI.

The expression of PARP-1 in HsiI cells (lane 3, upper panel) was observed to be less intense compared with the control HeLa cells (lane 2, upper panel) as shown in Figure 1(a). The expression of beta actin (internal control) was the same in HeLa and HsiI as shown in lanes 2 and 3 in the lower panel of the Figure 1(a). Densitometric analysis showed that about 66% reduction of expression of PARP-1 in HsiI cells. PARP-1 activity can be measured by determining NAD^+^ pool in cell extract since PARP-1 uses NAD^+^ as substrate [43]. Here, we observed practically no difference of NAD^+^ pool in HeLa and H-vector (negative control, HeLa transfected with vector without siRNA insert), implicating that transfection did not affect NAD^+^ content of cells. HsiI cells showed almost 37% elevated level of NAD^+^ pool (9.6 pmole/mg protein) as shown in Figure 1(b). This result indicated that reduced activity of PARP-1 was due to reduction of expression level of PARP-1.

### 3.2. Comet Assay to Detect DNA Breaks

**Figure 2:**
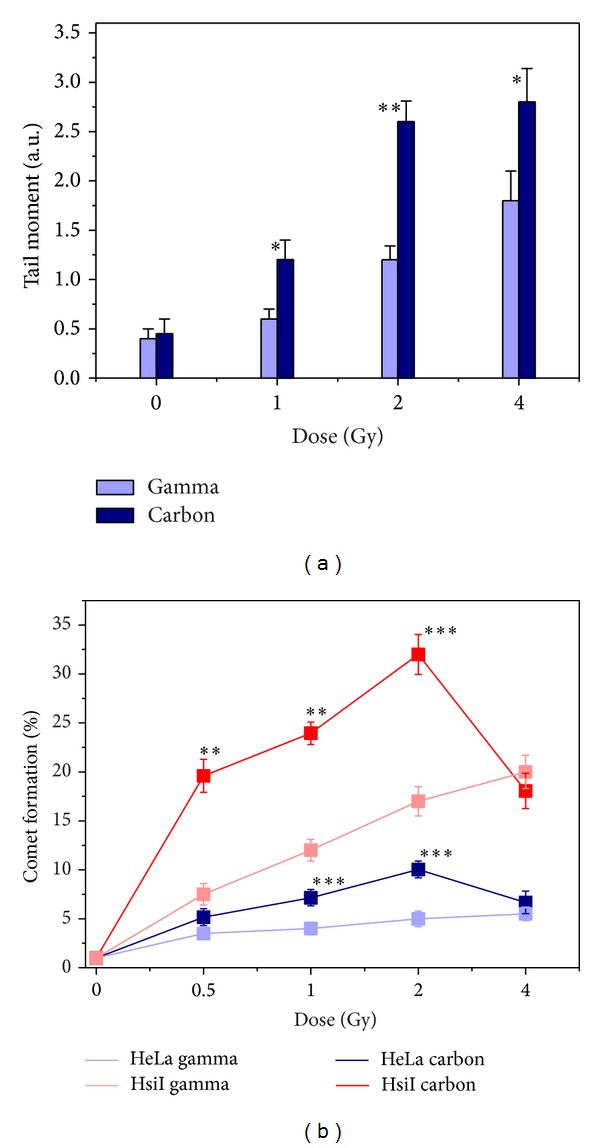
Comet Assay for DNA breaks detection after 24 h postirradiation of carbon ion beam and gamma radiation. (a) Tail moment was measured in HeLa cells after irradiation with carbon ion beam and gamma radiation at different doses (0–4 Gy). (b) % of comet formation in carbon ion beam and gamma irradiated each of both cells HeLa and HsiI at various doses (0–4 Gy). Statistical significance level was calculated within carbon ion beam and gamma irradiated groups of each cell type and denoted by *P* values. “∗” means (0.01 < *P* ≤ 0.05), “∗∗” means (0.001 < *P* ≤ 0.01), and “∗∗∗” means (*P* ≤ 0.001).

DNA damage by both carbon ion beam and gamma radiation was estimated by measuring tail moment of the comet produced and percentage of comet-producing cells. There was dose-dependent increase of tail moment of comet produced both by carbon ion beam and gamma radiation, but tail moment at each dose of carbon ion beam was significantly higher than that for gamma radiation as shown in Figure 2(a). Notably, there was no appreciable difference of tail moment at 1 Gy of gamma radiation with respect to unirradiated control, but carbon ion beam of same dose produced significantly higher tail moment compared with that for gamma radiation. Increase of tail moment with dose was very steep for carbon ion beam and reached maxima at 2 Gy, whereas that for gamma radiation was less steep. This data implicated that quality and quantity of DNA breaks were different for carbon ion and gamma. A large number of reports showed that high LET radiation such as carbon ion beam produced cluster DNA damage where huge number of DNA breaks was found in a very small region of DNA resulting higher number of DSBs [48–51]. The number of DNA breaks by low LET gamma in a particular region of DNA is far less than that of high LET radiation.

We also measured the percentage of comet forming cells by both types of irradiation as shown in Figure 2(b). The percentage of comet forming cell was higher for carbon ion beam compared with gamma in each type of cell at all doses except 4 Gy. For example, the percentage of HsiI cells that produced comet by gamma was 7.5 ± 1.1%, 12 ± 1.1%, and 17 ± 1.5% at dose 0.5 Gy, 1 Gy, and 2 Gy, respectively, whereas that by carbon ion beam was 19.6 ± 1.6%, 23.9 ± 1.1 %, and 32 ± 2.0%, respectively, at the corresponding doses. Furthermore, percentage of comet-producing HeLa cells after irradiation with carbon ion beam was also higher than that for gamma radiation as shown in Figure 2(b). However, there was no appreciable difference of comet-producing cells after irradiation with carbon ion beam or gamma radiation at as high as 4 Gy. This is evident from the data that cells become sensitized by knocking down of PARP-1 after irradiation with carbon ion beam and gamma radiation. A large number of reports showed that PARP-1 is involved in SSB DNA repair [1–3]. High LET radiation produces clustered DNA damage where high number of DSBs occurs in a small region of DNA. HsiI cells showed higher sensitivity than HeLa after irradiation with ^12^C, implicating that PARP-1 is also involved in DSB DNA repair pathway as evident from earlier reports [6, 7].

### 3.3. Clonogenic Cell Survival Assay after Irradiation with Carbon Ion Beam and Gamma Radiation

**Figure 3:**
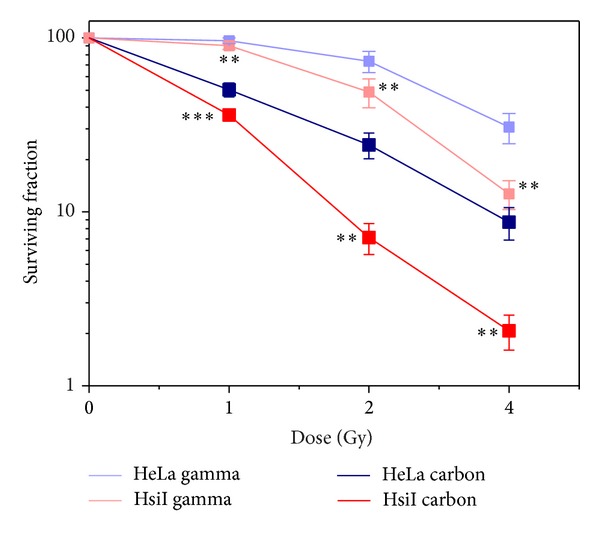
Surviving Fraction of HeLa and HsiI after irradiation with carbon ion beam and gamma radiation. Each point represents the mean with standard deviation. The significance level of difference of % survival at the same dose of both carbon ion beam and gamma radiation for each cell type was denoted by *P* values. “∗∗” means (0.001 < *P* ≤ 0.01) and “∗∗∗” means (*P* ≤ 0.001).

The dose-dependent decrease of % survival was observed after irradiation with both carbon ion beam and gamma radiation in each cell type, but higher killing was observed in carbon ion beam compared with the same dose of gamma radiation in each cell type as shown in Figure 3. This result implicated that recovery after DNA damage by carbon ion beam was significantly less than that of by gamma radiation. Cell killing of HeLa at 2 Gy and 4 Gy of carbon ion beam was ~4.2-fold (*P* = 0.006) and ~4-fold (*P* = 0.004) higher than that of gamma radiation. HeLa cells were radiosensitized by knocking down of PARP-1 (HsiI cells) and cell killing of HsiI at 2 Gy and 4 Gy was ~7-fold (*P* = 0.004) and ~7.5-fold (*P* = 0.004) higher, respectively, in carbon ion beam than gamma radiation. Therefore, radiosensitivity by inhibition of PARP-1 increased several folds when we used high LET carbon ion beam instead of low LET gamma radiation.

### 3.4. Cell Cycle Analysis

The cell cycle distribution in HeLa and HsiI cells irradiated with different doses of carbon ion beam and gamma radiation (0–4 Gy) was analyzed using ModFit LT software and percentage of cell population in different phases was obtained. The mean of % cell population with standard deviation at each phase obtained from three independent experiments was given in the Table 1. The *P* values were calculated at each dose with respect to unirradiated control and shown within the brackets. Significant G_2_/M arrest was observed for both carbon ion beam and gamma radiation at 4 Gy, but increase of cell population at G_2_/M phase was almost 100% and 26% in HeLa with respect to unirradiated control for carbon ion beam and gamma radiation, respectively. In case of HsiI cells, the increase of G_2_/M cell population with respect to unirradiated control was ~141% and ~80% for carbon ion beam and gamma radiation, respectively. Earlier reports also showed ionizing radiation induced G_2_/M arrest [12, 52–54]. Here, we observed that both the high LET and low LET triggered G_2_/M arrest, but the intensity as determined by cell population at G_2_/M was higher for high LET.

Takahisa et al. showed the S-phase delay and subsequent G_2_/M arrest in human pancreatic cancer cell line after irradiation with both gamma radiation and LET 13 and LET 70 carbon ion beam in presence of PARP inhibitor [55]. But in our present work, there was as such no difference in cell population in S-phase after irradiation with gamma radiation in HeLa or PARP-1 knocked down cells HsiI whereas significant (*P* = 0.013) increase (~23% with respect to unirradiated control) of S-phase cell population was observed at as low as 0.5 Gy of carbon ion beam in HeLa. This data implicated that DNA damage and subsequent signal induced after irradiation with carbon ion beam are qualitatively and/or quantitatively different from those of gamma radiation. Possibly, the clustered DNA lesions which produce huge number of DSB affect severely the replication process. Furthermore, S-phase delay in HsiI cells was not observed up to 1 Gy of carbon ion beam but observed at as high as 2 Gy. We do not know the exact reason behind it. Generally, DSB repair by homologous recombination (HR) pathway occurs in late S-phase [56, 57] and few earlier studies showed the involvement of PARP-1 in HR pathway [26, 58, 59]. Hence we observed S-phase delay in HeLa, where PARP-1 is fully active and most likely involved in DSB repair by HR pathway after irradiation with carbon ion beam.

### 3.5. Apoptosis Induction as Detected by Nuclear Fragmentation and Caspase-3 Activity after Irradiation with Carbon Ion Beam and Gamma Radiation

**Figure 4:**
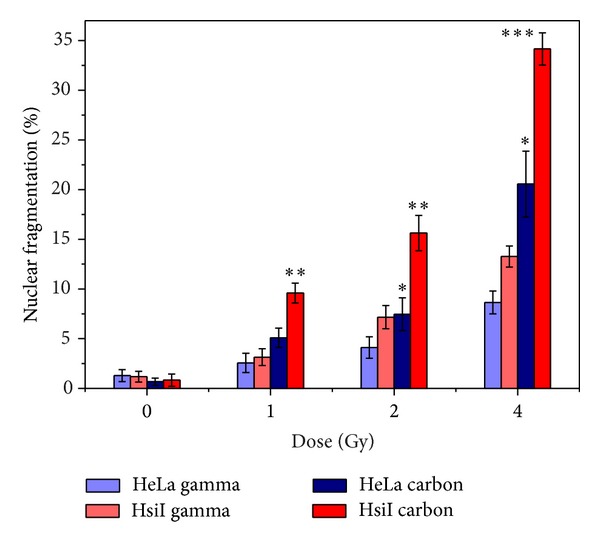
Induction of apoptosis detected by nuclear fragmentation after irradiation with carbon ion beam and gamma radiation in HeLa and HsiI cells after 24 h incubation. Each bar represents mean % nuclear fragmentation with standard deviation in respective cell types. *P* values at each dose of both carbon ion beam and gamma radiation for each cell type were calculated using 2-tailed paired-samples* t*-test and denoted by “∗” (0.01 < *P* ≤ 0.05), “∗∗” (0.001 < *P* ≤ 0.01), and “∗∗∗” (*P* ≤ 0.001). *P* values greater than 0.05 were left blank.

**Figure 5:**
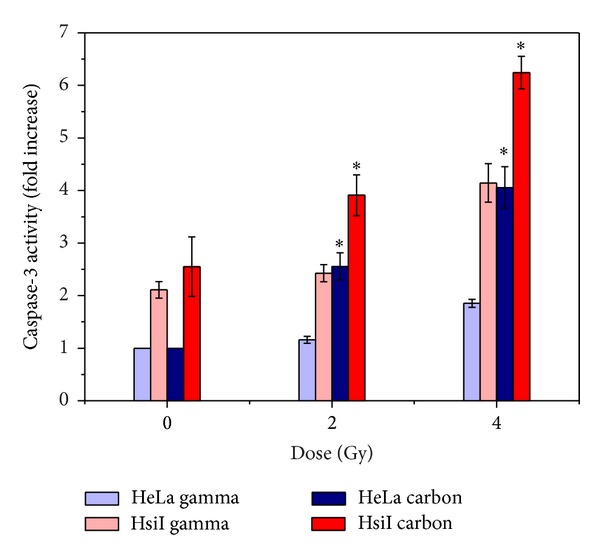
Caspase-3 activity in HeLa and HsiI cells irradiated with carbon ion beam and gamma radiation. Each histogram represents the mean of fold increase with respect to unirradiated control HeLa of 3 independent experiments with the standard deviations (vertical lines). The significance values were obtained in carbon ion beam compared with gamma radiation at a particular dose in each cell type and denoted as “∗” (0.01 < *P* ≤ 0.05).

Irradiation with carbon ion beam and gamma radiation increased the nuclear fragmentation as detected by the fragmented nucleus under fluorescence microscope after staining with Hoechst dye in HeLa and HsiI cells in a dose-dependent manner (0–4 Gy). The results were shown in Figure 4. Induction of apoptosis by carbon ion beam at each dose was observed to be always higher than that of gamma radiation in both HeLa and HsiI cells. For example, nuclear fragmentation by carbon ion beam was significantly higher than gamma radiation at and above 2 Gy in HeLa. Notably, carbon ion beam induced significantly higher nuclear fragmentation than gamma radiation at all doses used. These observations implicated that apoptotic cell death by carbon ion beam was significantly higher than gamma radiation in both HeLa and HsiI although the extent of induction of apoptosis was much higher in HsiI cells. We also observed dose-dependent (0–4 Gy) increase of caspase-3 activity in HeLa and HsiI cells after irradiation with both carbon ion beam and gamma radiation. Increase of caspase-3 activity by carbon ion beam was significantly higher (almost 2-fold) than that of gamma radiation in HeLa cells as shown in Figure 5. HsiI cells also showed the similar type of response with greater extent of apoptosis.

We did not check necrotic cell death, if any, after irradiation, but we measured induction of apoptosis as detected by nuclear fragmentation and caspase-3 activation. Induction of apoptosis was significantly higher for carbon ion beam compared with gamma in both HeLa and HsiI cells, but later cell type showed more pronounced effect. We observed that activity of caspase-3 was significantly higher in unirradiated HsiI cells compared with that of unirradiated HeLa cells (Figure 5). The reason behind such higher activation of caspase-3 upon inhibition of PARP-1 in HsiI cells is not clear to us. We observed earlier that inhibition of PARP by benzamide resulted in release of cytochrome c in Chinese hamster V79 cells [30]. But we do not know the status of cytochrome c in our experimental conditions. In any way, either cytochrome c-dependent or -independent way, caspase-3 activation is high in HsiI cells and possibly that is why induction of apoptosis as well as cell death as detected by clonogenic survival assay showed always in higher extent in HsiI cells compared with HeLa after irradiation with high or low LET radiation. Carbon ion beam produced higher cell killing than gamma in both HeLa and HsiI although the later cell type showed greater effect. Possibly, that is the reason why carbon ion beam therapy is becoming so much popular over conventional radiotherapy such as low LET gamma or X-ray in killing radioresistant tumor cells. PARP-1 inhibitor has long been used as radiosensitizer [32–35], but PARP-1 inhibition sensitized the cells several folds when carbon ion beam is used instead of gamma radiation. Therefore, heavy ion beam along with PARP-1 inhibition could be more effective in killing cancer cells.

## 4. Conclusions

DNA damage and subsequent cascades of signaling are different for high LET carbon ion beam and low LET gamma radiation, especially the high LET carbon ion beam can interfere the replication process as detected by S-phase delay while the low LET has no effect on S-phase in our experimental condition. Cell killing and apoptosis induction by high LET carbon ion beam were higher than those by low LET gamma in both HeLa and PARP-1 knocked down cells (HsiI). Radiosensitization upon knocking down of PARP-1 gene was several folds greater for high LET carbon ion beam than low LET gamma radiation. So, radiotherapy in combination with PARP-1 inhibition would be more effective when high LET such as carbon ion beam is used instead of low LET gamma radiation.

## Supplementary Material


*Apoptosis Induction as Detected by Nuclear Fragmentation*. We showed that carbon ion beam irradiation induced more nuclear fragmentation than gamma radiation in both HeLa and HsiI cells (PARP-1 knocked down cells) as discussed in the main manuscript. Furthermore, HsiI cells were found to have more number of fragmented nuclei than HeLa for both types of radiation as shown in Figure 4 in the main manuscript. Here, the typical photographs of undamaged nucleus and the nucleus irradiated with 4 Gy of carbon ion beam in HsiI cells are shown in Figure S1 (a) & S1 (b) respectively.
*Apoptosis Induction as Detected by Caspase-3 Activity Assay.* Induction of apoptosis after irradiation with carbon ion beam and gamma was measured by the fold increase of caspase-3 activity in both HeLa and HsiI cells with compared to unirradiated HeLa cells as shown in Figure 5 in the main manuscript. Here, we have also plotted the same findings as % caspase-3 activity in Figure S2. The caspase-3 activity in unirradiated HeLa was taken as 100% caspase-3 activity and accordingly rest were calculated and plotted. Our data showed that carbon ion beam induced caspase-3 activity was always significantly higher than the gamma induced caspase-3 activity in a particular cell type.

## Figures and Tables

**Table 1:** Cell cycle distribution in HeLa and HsiI cells by FACS. Cells were irradiated with different doses of carbon ion beam and gamma radiation (0–4 Gy). The percentages of cell cycle distribution were evaluated by ModFit LT software using PI staining. Values represent the mean ± SD of three independent experiments. *P* values were calculated at 0.05 confidence level with respect to unirradiated control for each phase of each dose and shown within the brackets.

Dose (Gy)	Irradiated with gamma						Irradiated with carbon					
	HeLa (% of cell population with standard deviation)			HsiI (% of cell population with standard deviation)			HeLa (% of cell population with standard deviation)			HsiI (% of cell population with standard deviation)		
	G_0_/G_1_	S	G_2_/M	G_0_/G_1_	S	G_2_/M	G_0_/G_1_	S	G_2_/M	G_0_/G_1_	S	G_2_/M
0	24.57 ± 0.33	59.77 ± 1.09	15.64 ± 0.80	19.46 ± 0.84	69.79 ± 1.89	10.74 ± 1.04	40.77 ± 0.36	43.87 ± 1.08	15.35 ± 0.72	37.33 ± 0.07	47.46 ± 0.38	15.19 ± 0.40

0.5	27.64 ± 0.17 (0.000)	57.35 ± 0.70 (0.114)	15.00 ± 0.62 (0.727)	21.05 ± 0.55 (0.041)	65.40 ± 1.48 (0.008)	13.54 ± 1.08 (0.013)	28.49 ± 0.16 (0.000)	53.58 ± 0.82 (0.013)	17.92 ± 0.78 (0.529)	34.30 ± 0.80 (0.009)	48.85 ± 1.58 (0.846)	16.84 ± 0.87 (0.510)

1.0	27.73 ± 0.55 (0.000)	56.65 ± 0.34 (0.038)	15.61 ± 0.40 (1.000)	26.54 ± 0.64 (0.000)	54.77 ± 0.90 (0.000)	18.68 ± 0.33 (0.000)	26.89 ± 0.27 (0.000)	57.57 ± 0.94 (0.001)	15.53 ± 1.11 (1.000)	30.69 ± 1.29 (0.000)	46.68 ± 1.27 (0.975)	22.62 ± 0.89 (0.000)

2.0	29.28 ± 0.65 (0.000)	53.72 ± 1.22 (0.000)	17.00 ± 0.57 (0.180)	24.12 ± 0.67 (0.000)	56.76 ± 1.40 (0.000)	19.11 ± 0.76 (0.000)	32.38 ± 1.22 (0.000)	55.87 ± 2.27 (0.003)	11.74 ± 1.11 (0.268)	19.16 ± 0.48 (0.000)	58.12 ± 0.71 (0.000)	22.71 ± 0.52 (0.000)

4.0	20.8 ± 0.90 (0.000)	59.66 ± 2.11 (1.000)	19.53 ± 1.27 (0.000)	20.16 ± 0.42 (0.509)	62.44 ± 0.68 (0.000)	17.39 ± 1.10 (0.000)	23.59 ± 1.50 (0.000)	47.39 ± 6.57 (0.501)	29.01 ± 5.08 (0.000)	34.16 ± 1.34 (0.007)	22.71 ± 4.32 (0.000)	43.12 ± 3.07 (0.000)
