# Supplementary material for: Radiosensitivity and Induction of Apoptosis by High LET Carbon Ion Beam and Low LET Gamma Radiation: A Comparative Study
Source: Scientifica (Cairo). 2014 Jun 15;2014:438030. doi: 10.1155/2014/438030 (PMC4083825; doi:10.1155/2014/438030)
Supplement: Supplementary file 1 — Apoptosis Induction as Detected by Nuclear Fragmentation. We showed that carbon ion beam irradiation induced more nuclear fragmentation than gamma radiation in both HeLa and HsiI cells (PARP-1 knocked down cells) as discussed in the main manuscript. Furthermore, HsiI cells were found to have more number of fragmented nuclei than HeLa for both types of radiation as shown in Figure 4 in the main manuscript. Here, the typical photographs of undamaged nucleus and the nucleus irradiated with 4 Gy of carbon ion beam in HsiI cells are shown in Figure S1 (a) & S1 (b) respectively. Apoptosis Induction as Detected by Caspase-3 Activity Assay. Induction of apoptosis after irradiation with carbon ion beam and gamma was measured by the fold increase of caspase-3 activity in both HeLa and HsiI cells with compared to unirradiated HeLa cells as shown in Figure 5 in the main manuscript. Here, we have also plotted the same findings as % caspase-3 activity in Figure S2. The caspase-3 activity in unirradiated HeLa was taken as 100% caspase-3 activity and accordingly rest were calculated and plotted. Our data showed that carbon ion beam induced caspase-3 activity was always significantly higher than the gamma induced caspase-3 activity in a particular cell type. [file 438030.f1.pdf]

Supplementary Figures:

Apoptosis Induction as Detected by Nuclear Fragmentation. We showed that carbon ion beam irradiation induced more nuclear fragmentation than gamma radiation in both HeLa and HsiI cells (PARP-1 knocked down cells) as discussed in the main manuscript. Furthermore, HsiI cells were found to have more number of fragmented nuclei than HeLa for both types of radiation as shown in Figure 4 in the main manuscript. Here, the typical photographs of undamaged nucleus and the nucleus irradiated with 4 Gy of carbon ion beam in HsiI cells are shown in Figure S1(a) & S1(b) respectively.

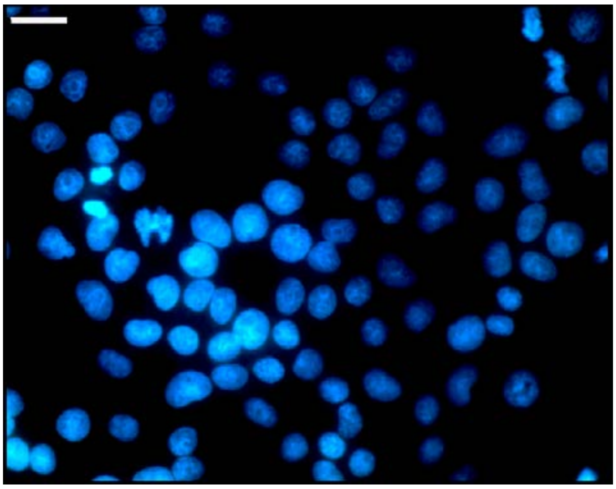

Figure S1 (a)

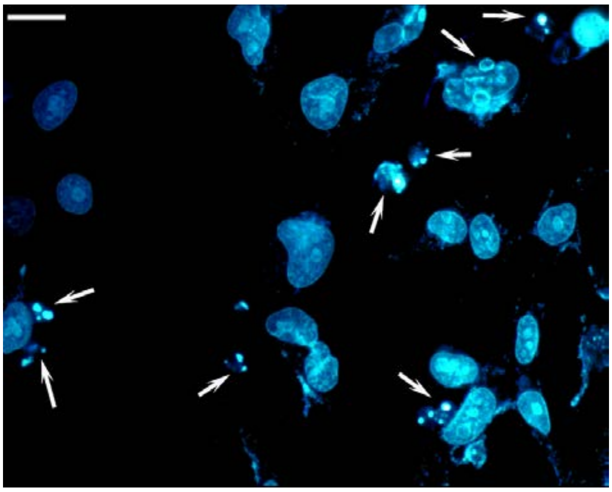

Figure S1 (b)

FIGURE S1: Detection of apoptosis by nuclear fragmentation. Scale bar represents 20 µm. (a) A typical photograph of un-irradiated HsiI cells stained with Hoechst dye is shown. (b) A typical photograph of HsiI cells irradiated with 4 Gy of carbon ion beam followed by 24 h incubation and stained with Hoechst dye is shown here. Arrows indicate fragmented nucleus or apoptotic bodies.

Apoptosis Induction as Detected by Caspase-3 Activity Assay. Induction of apoptosis after irradiation with carbon ion beam and gamma was measured by the fold increase of caspase-3 activity in both HeLa and HsiI cells with compared to unirradiated HeLa cells as shown in Figure 5 in the main manuscript. Here, we have also plotted the same findings as % caspase-3 activity in Figure S2. The caspase-3 activity in unirradiated HeLa was taken as 100% caspase-3 activity and accordingly rest were calculated and plotted. Our data showed that carbon ion beam induced caspase-3 activity was always significantly higher than the gamma induced caspase-3 activity in a particular cell type.

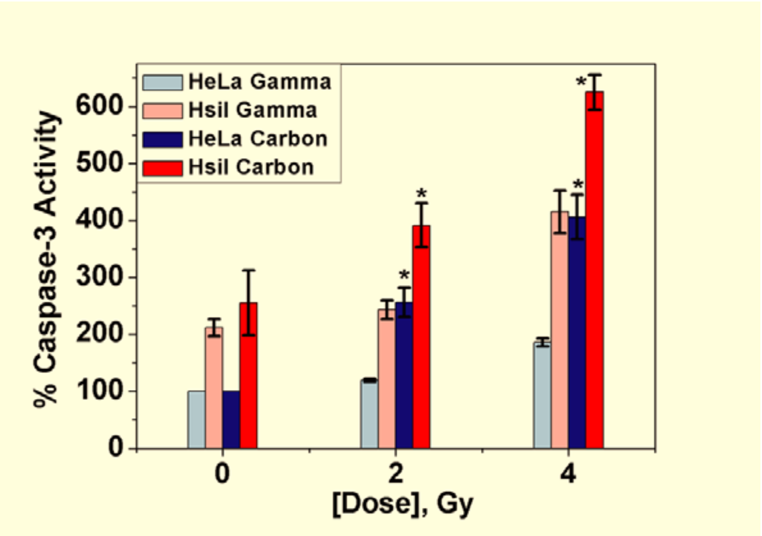

FIGURE S2: % Caspase-3 activity in HeLa and HsiI cells treated with carbon ion beam and gamma radiation. Each histogram represents the mean of % caspase-3 activity with respect to un-irradiated control HeLa of 3 independent experiments with the standard deviations (vertical lines). The significance values were obtained in carbon ion beam compared with gamma radiation at a particular dose in each cell type and denoted as “*” ($0.01 < P \leq 0.05$).
